# Inability to predict postpartum hemorrhage: insights from Egyptian intervention data

**DOI:** 10.1186/1471-2393-11-97

**Published:** 2011-11-28

**Authors:** Ndola Prata, Sabry Hamza, Suzanne Bell, Deborah Karasek, Farnaz Vahidnia, Martine Holston

**Affiliations:** 1 Bixby Center for Population, Health and Sustainability, School of Public Health, University of California at Berkeley, 229 Warren Hall, UC-Berkeley, Berkeley, CA 94720-7360, USA; 2 Obstetrics and Gynecology Department, Faculty of Medicine, Ain Shams University, Abbasia, Cairo, Egypt; 3 Venture Strategies Innovations, 2115 Milvia Street Suite 4A, Berkeley, CA 94704, USA

## Abstract

**Background:**

Knowledge on how well we can predict primary postpartum hemorrhage (PPH) can help policy makers and health providers design current delivery protocols and PPH case management. The purpose of this paper is to identify risk factors and determine predictive probabilities of those risk factors for primary PPH among women expecting singleton vaginal deliveries in Egypt.

**Methods:**

From a prospective cohort study, 2510 pregnant women were recruited over a six-month period in Egypt in 2004. PPH was defined as blood loss ≥ 500 ml. Measures of blood loss were made every 20 minutes for the first 4 hours after delivery using a calibrated under the buttocks drape. Using all variables available in the patients' charts, we divided them in ante-partum and intra-partum factors. We employed logistic regression to analyze socio-demographic, medical and past obstetric history, and labor and delivery outcomes as potential PPH risk factors. Post-model predicted probabilities were estimated using the identified risk factors.

**Results:**

We found a total of 93 cases of primary PPH. In multivariate models, ante-partum hemoglobin, history of previous PPH, labor augmentation and prolonged labor were significantly associated with PPH. Post model probability estimates showed that even among women with three or more risk factors, PPH could only be predicted in 10% of the cases.

**Conclusions:**

The predictive probability of ante-partum and intra-partum risk factors for PPH is very low. Prevention of PPH to all women is highly recommended.

## Background

### Introduction

Primary postpartum hemorrhage (PPH) is the single largest contributor to maternal mortality worldwide. Throughout Africa and Asia, hemorrhage accounts for 30% or more of all maternal deaths, most of which is PPH [1]. Even if effective antenatal screening existed, hemorrhage often occurs in women with no identifiable ante-partum risk factors. Risk factors in the intra-partum period may provide opportunity for intervention, or for active management in the early stages of PPH.

Despite the severe burden of PPH, few studies have examined risk factors predicting PPH in developing countries. The primary objective of this study is to investigate the ability to predict PPH by early screening of women with selected risk factors. We hypothesize that a combination of ante-partum and intra-partum risk factors will be associated with incidence of PPH, and that these factors will have utility for screening and clinical management of labor. The secondary objective of this study is to identify the components of active management of the third stage of labor (AMTSL) that have the greatest impact on PPH incidence. AMSTL is a component of internationally accepted clinical protocol for obstetric management consisting of four components: use of uterotonics, uterine massage, early cord clamping, and cord traction. The importance of AMSTL in PPH is well established, yet the relative importance of each component of the process has not been examined.

### Previous Research

Previous research has identified demographic, ante-partum, and intra-partum risk factors that are associated with risk of hemorrhage, but these associations are not predictive. In addition, the majority of these studies focus on women in the United States or Europe who may face different delivery circumstances from women in developing countries. An extensive literature review on PubMed that investigated ante-partum and intra-partum risk factors for PPH was carried out. All of the risk factors found are described below; citations are only for the most recent article published on the topic.

Investigators have established myriad demographic, ante-partum, and intra-partum risk factors. Demographic risk factors associated with excessive blood loss (greater than 500 or 1000 ml) in the first 24 hour after vaginal delivery include non-white ethnicity [2], and older age [3]. Among ante-partum risk factors, history of PPH [4], history of blood disorders [2], nulliparity [5], low parity [6], grand multiparty [7], high blood pressure [8], ante-partum hemorrhage [9], multiple pregnancy [3], fewer than four prenatal visits [10], lack of iron supplementation/anemia [3], over distended uterus [5], pregnancy induced hypertension [11], and increased maternal BMI [12] have been significantly associated with PPH in previous studies. Intra-partum risk factors may indicate a different point of intervention than ante-partum factors. Past studies have shown preterm birth [7], episiotomy or genital tract trauma [11], labor augmentation or instrumental delivery [8], use of oxytocics for induction or augmentation [7], induction of labor [8], abnormal fetal presentation [2], manual removal of placenta or retained placenta [11], uterine atony [4], coagulopathy [13], placenta previa or accreta [14], perineal tear [15], intra-partum hemorrhage [5], non-use of oxytocics after delivery [16], birthweight [10], placental weight [7], and perinatal death [17] to be significantly associated with PPH.

Prolonged labor is consistently associated with PPH as well, although studies differ on a meaningful threshold for each stage. Stones et al. associated labor of over 12 hours with increased risk of PPH [18]. While multiple studies associate prolonged 1^st ^and 2^nd ^stage of labor with increased risk of PPH, the magnitude of association for the 3^rd ^stage appears to be pronounced [2,5,7-9,11,14,19-26].

Despite all that is known about PPH risk factors, risk factors associated with *primary *PPH are not useful in predicting patients who will continue to bleed after first-line therapy [27]. And while previous studies have identified significant associations among ante-partum and intra-partum risk factors, Tsu's paper (1993) is the only published work examining predictive probability of screening for PPH. While predictive values were less than 7%, she found that screening tests for maternal height, parity, and obstetric history could identify one third of PPH cases [28].

## Methods

We conducted a secondary data analysis using data from prospective, hospital based operations research in Egypt in 2006. The objective of the original operations research was to compare the current protocol for the AMTSL with the use of 600 μg of misoprostol. The conclusion was that misoprostol should be considered for inclusion in the AMTSL protocol where oxytocin and or ergometrine are not consistently and appropriately used during the 3^rd ^stage of labor. The original study was analyzed as a pre-/post-intervention study. In the current study, we analyzed all women who experienced PPH as 'cases' and all women who did not experience PPH as 'controls.' Details on design data, collection, and results of the original study are reported elsewhere [29]. Over a 6-month period, 2532 pregnant women were recruited into the study from three university hospitals. Inclusion criteria were: anticipated singleton vaginal delivery, gestational age greater than 36 weeks, and ability to give informed consent. Women who delivered by cesarean section or women with missing information on delivery type were excluded from this analysis, leaving a total of 2510 women.

The primary outcome of interests was incidence of PPH. In accordance with international clinical protocol, PPH was defined as blood loss greater than 500 ml during the first 4 hours after delivery. Following delivery and the clamping of the umbilical cord, a calibrated drape was placed under the women's buttocks for blood loss measurement. This drape remained under the women's buttocks for 4 hours, during which time blood loss was read cumulatively every 20 minutes. Total blood loss was established after the bleeding had stopped. In the original study, women in the misoprostol group were 70% less likely to bleed 500 ml or more (OR = 0.30, 95% CI 0.16-0.56) compared to those in the current practices group. In addition, the women in the misoprostol group needed additional interventions because of postpartum hemorrhage only 11% of the time, compared to 73% among the current practices group.

To evaluate the value of risk factors for screening and labor management, attending physicians collected both ante-partum and intra-partum information using questionnaires that were completed at the time of admission, during labor, after delivery for up to 4 hours, and before discharge. Variables collected at the time of admission for delivery include maternal age (30 years or older), education (literacy), nulliparity, previous antenatal care (no care or high number of visits compared), PPH in a previous pregnancy, history of obstetric complications, intact membranes, anemia, and cervical dilation. Anemia was measured by blood collected after enrollment, and defined as ante-partum hemoglobin at or below 11 mg/dl.

Intra-partum variables include vaginal delivery with instruments, episiotomy, labor augmentation, retained placenta, vaginal tears, fetal macrosomia (> 3500 g), length of 1^st ^and 2^nd ^stage, and absence of AMTSL. Additionally, the absence of each component of AMTSL was measured separately, including use of uterotonics in the 3^rd ^stage, early cord clamping, cord traction, and uterine massage.

Ethical review for the study was provided by the Committee for the Protection of Human Subjects at the University of California, Berkeley and the Egyptian ethics committees. The Healthy Mother Healthy Child Project, which was implemented by John Snow Inc. under United States Agency for International Development (USAID) Contract No. 263-C-00-98-0041-00, was responsible for study implementation. Venture Strategies for Health and Development (VSHD) donated the misoprostol used in the operations research (Misotac; Sigma Co., Moubarak industrial City, First Quarter Quesna, Egypt).

Data was entered using Epi-Info version 2002 (Centers for Disease Control and Prevention, Atlanta, Georgia, USA), and statistical analysis was performed with the STATA^® ^version 8.0 (StataCorp, LP, College Station, Texas, USA). We constructed separate, multiple logistic regression models for ante-partum and intra-partum variables associated with PPH, but not as a stepwise regression. Model 1 included demographic and antepartum variables; Model 2 included intra-partum variables and one variable to represent AMTSL; Model 3 is a variation of Model 2 with AMTSL separated out by each component; and the final model, Model 4, was the combination of demographic/antepartum, intra-partum, and separated AMTSL factors. Separating each of the four components of AMTSL allowed us to assess the relative importance of each component. Odds ratios were provided as the measure of effect. Results from the final model, Model 4, were used in post-model estimation of predictive probability of PPH. In addition, we created cumulative scores based on the number of risk factors (1, 2, 3, or 4+). Sensitivity and specificity were calculated for the cumulative risk factor probabilities and for the combined measure of AMTSL. Receiver operating curves (ROC) were derived from these sensitivity and specificity estimates. Significant associations were established at p-values < 0.05.

## Results

The incidence of PPH among the 2510 singleton vaginal deliveries was 3.71% (n = 93). The distribution of demographics, ante-partum, and intra-partum risk factors are listed in Table 1. Odds ratios from bivariable analyses of risk factors and incidence of PPH are reported along with associated p-values and 95% confidence intervals (CIs). Among unadjusted ante-partum variables, history of PPH in a previous pregnancy increased the risk of PPH by almost 69 times. Women who had one antenatal care (ANC) visit, those with no care, and those with higher levels of care also had an increased risk of PPH. No other ante-partum risk factors were significant in bivariable logistic regressions.

**Table 1:** Distribution of ante-partum and intra-partum factors and crude odds ratios associated with PPH (blood loss ≥500 mL) for vaginal deliveries

	PPH	No PPH	OR	p-value	95% CI
	93 N (%)	2417 N (%)			
**Ante-Partum**					
Age					
< 30	72 (77.4)	1,892 (79.4)	1.00		
30 +	21 (22.6)	492 (20.6)	1.12	0.650	(0.68, 1.84)
Education					
illiterate	34 (37.8)	1,092 (45.8)	1.00		
literate	56 (62.2)	1, 295 (54.2)	1.39	0.137	(0.90, 2.14)
Parity					
multiparous	53 (61.6)	1,524 (64.85)	1.00		
nuliparous	33 (38.4)	826 (35.15)	1.15	0.539	(0.74, 1.79)
Antenatal care					
1 visit	2 (2.3)	275 (12.0)	1.00		
No ANC	29 (33.3)	874 (38.1)	4.56	0.039	(1.08, 19.24)
2-3 visits	21 (24.1)	548 (23.9)	5.27	0.025	(1.22, 22.63)
4+ visits	35 (40.2)	597 (26.0)	8.06	0.004	(1.93, 33.76)
History of PPH					
None	88 (94.6)	2,415 (99.9)	1.00		
Prior PPH	5 (5.4)	2 (0.1)	68.61	< 0.001	(13.13, 358.51)
Previous obstetric complications					
none	92 (98.9)	2,306 (95.4)	1.00		
Other non-PPH	1 (1.1)	111 (4.6)	0.23	0.141	(0.03, 1.63)
Membranes					
ruptured	24 (26.1)	614 (25.7)	1.00		
intact	68 (73.9)	1,775 (74.3)	0.98	0.934	(0.61, 1.57)
Anemia					
Ante-partum hemoglobine> 11 mg/dl	55 (74.3)	1,332 (77.8)	1.00		
Ante-partum hemoglobine < = 11 mg/dl	19 (25.7)	380 (22.2)	1.21	0.480	(0.71, 2.07)
Cervical dilation on admission					
up to 3 cm	4 (4.3)	161 (6.7)	1.00		
4-7 cm	57 (62.0)	1,548 (64.8)	1.48	0.453	(0.53, 4.14)
> 7 cm	31 (33.7)	680 (28.5)	1.83	0.260	(0.64, 5.27)
**Intra-partum**					
Delivery					
Spontaneous delivery	92 (98.9)	2,411 (99.8)	1.00		
vaginal delivery with instruments	1 (1.1)	6 (0.2)	4.37	0.174	(0.52, 36.65)
Episiotomy					
no	34 (37.0)	1,328 (55.8)	1.00		
yes	58 (63.0)	1,052 (44.2)	2.15	< 0.001	(1.40, 3.31)
Labor augmentation					
no	44 (47.3)	1,562 (64.7)	1.00		
yes	49 (52.7)	854 (35.3)	2.04	0.001	(1.34, 3.09)
Complete placenta expulsion					
yes	92 (98.9)	2,395 (99.5)	1.00		
no	1 (1.1)	13 (0.5)	2.00	0.506	(0.26, 15.47)
Vaginal tears					
no	82 (89.1)	2,216 (95.3)	1.00		
yes	10 (10.9)	110 (4.7)	2.46	0.010	(1.24, 4.87)
Fetal weights					
< 3500 mg	54 (59.3)	1,692 (71.7)	1.00		
Macrosomia (> 3500 mg)	37 (40.1)	667 (28.3)	1.74	0.011	(1.13, 2.67)
Length of 1st and 2nd stage					
< 1 hour	10 (11.0)	634 (26.7)	1.00		
1 - 2.9 hours	27 (29.7)	670 (28.2)	2.55	0.012	(1.23, 5.32)
3 - 4.9 hours	34 (37.3)	629 (26.5)	3.43	0.001	(1.68, 7.00)
5 + hours	20 (22.0)	443 (18.6)	2.86	0.007	(1.33, 6.17)
AMTSL					
Yes	62 (66.7)	2,281 (94.4)	1.00		
No	31 (33.3)	136 (5.63)	8.39	< 0.001	(5.27, 13.35)
AMTSL components					
Use of uterotonics in 3rd stage					
yes	78 (83.9)	2,333 (96.6)	1.00		
no	15 (16.1)	83 (3.4)	5.40	< 0.001	(2.98, 9.79)
Early cord clamping					
yes	86 (92.5)	2,376 (98.3)	1.00		
no	7 (7.5)	41 (1.7)	4.72	< 0.001	(2.06, 10.82)
Cord traction					
yes	85 (91.4)	2,399 (99.3)	1.00		
no	8 (8.6)	18 (0.7)	12.54	< 0.001	(5.31, 29.66)
Uterine massage					
yes	82 (88.2)	2,383 (98.6)	1.00		
no	11 (11.8)	34 (2.4)	9.40	< 0.001	(4.60, 19.21)

Among intra-partum risk factors, episiotomy, labor augmentation, vaginal tears, macrosomia, and length of the first and second stage of labor were each associated with an increased odds of PPH. Women who did not receive AMTSL were 8 times more likely to have PPH. When assessed separately, omission of each component of AMTSL was also significantly associated with PPH risk.

Table 2 shows separate adjusted multivariable models for ante-partum risk factors (Model 1), intra-partum risk factors, (Models 2 and 3) and a full model (Model 4) containing both sets of variables. Components of AMTSL were included separately (Model 3) and together (Model 2) in intra-partum models. Each AMTSL component was considered individually in the full model. History of previous PPH (OR = 105.44, 95% CI 15.14-734.53), 2-3 ANC visits (OR = 7.02, 95% CI 1.49-33.01), and over 4 ANC visits (OR = 10.63, 95% CI 2.30-49.03) each remained positively associated with increased odds of PPH in the ante-partum only model. Additionally, a multivariable intra-partum model that considered the four components of AMTSL combined showed that labor augmentation with oxytocin (OR = 1.77, 95% CI 1.11-2.81), prolonged labor (1-2.9 hours OR = 2.42, 95% CI 1.15-5.06; 3-4.9 hours OR = 3.25, 95% CI 1.62-6.53; 5+ hours OR = 2.66, 95% CI 1.22-5.82), and AMTSL (OR = 105.44, 95% CI 15.14-734.53) were significantly associated with risk of PPH. When each component of AMTSL was included separately, fetal macrosomia (OR = 1.59, 95% CI 1.01-2.50) became marginally significant. In this model, use of uterotonics during the 3^rd ^stage of labor was the only AMTSL component associated with PPH (OR = 9.74, 95% CI 4.58-20.77). Episiotomy was marginally associated with PPH risk (p = 0.08). Vaginal tears, uterine massage, cord traction, and early cord clamping, though significant in the unadjusted analysis, were not associated with PPH after controlling for other intra-partum risk factors.

**Table 2:** Adjusted multivariable logistic regression models for ante-partum and intra-partum risk factors associated with PPH (blood loss ≥500 mL) for vaginal deliveries

	Model 1		Model 2		Model 3		Model 4	
	antepartum		intra-partum		AMTSL		Full model	
	**OR for PPH**	**95% CI**	**OR for PPH**	**95% CI**	**OR for PPH**	**95% CI**	**OR for PPH**	**95% CI**

**Ante-Partum**								
Age								
< 30	1.00		-		-		1.00	
30 +	1.47	(0.75, 2.88)	-		-		2.04	(0.92, 4.52)
Education								
illiterate	0.83		-		-		1.00	
literate	1.00	(0.39, 1.79)	-		-		0.62	(0.28, 1.42)
Parity								
multiparous	1.21		-		-		1.00	
nuliparous	1.00	(0.67, 2.19)	-		-		0.84	(0.40, 1.78)
Antenatal care								
1 visit	1.00		-		-		1.00	
No ANC	2.67	(0.58, 12.32)					1.61	(0.30, 8.51)
2-3 visits	7.02	(1.49, 33.01)					5.89	(1.13, 30.62)
4+ visits	10.63	(2.30, 49.03)	-		-		7.43	(1.44, 38.46)
History of PPH								
None	1.00		-		-		1.00	
Prior PPH	105.44	(15.14, 734.53)	-		-		305.14	(48.02, 1938.87)
Previous obstetric complications								
none	1.00		-		-		1.00	
prior non-PPH complications	0.26	(0.04, 1.84)	-		-		0.25	(0.02, 3.80)
Membranes								
ruptured	1.00		-		-		1.00	
intact	1.35	(0.73, 2.50)	-		-		1.06	(0.54, 2.09)
Anemia								
Ante-partum hemoglobine> 11 mg/dl	1.00		-		-		1.00	
Ante-partum hemoglobine < = 11 mg/dl	1.76	(0.98, 3.16)	-		-		2.73	(1.43, 5.23)
Cervical dilation on admission								
up to 3 cm	1.00		-		-		1.00	
4-7 cm	3.62	(0.51, 25.77)	-		-		4.60	(0.70, 30.38)
> 7 cm	3.67	(0.47, 28.62)	-		-		6.34	(0.87, 45.94)
**Intra-partum**								
Delivery								
Spontaneous delivery	-		1.00		1.00		1.00	
vaginal delivery with instruments	-		1.37	(0.13, 14.22)	1.35	(0.11, 15.87)	3.59	(0.35, 36.79)
Episiotomy								
no	-		1.00		1.00		1.00	
yes	-		1.60	(0.97, 2.63)	1.55	(0.94, 2.56)	1.72	(0.69, 4.30)
Labor augmentation								
no	-		1.00		1.00		1.00	
yes	-		1.77	(1.11, 2.81)	1.94	(1.17, 3.20)	2.37	(1.14, 4.93)
Complete placenta expulsion								
yes	-		1.00		1.00		1.00	
no	-		1.32	(0.55, 3.16)	2.91	(0.56, 15.07)	21.68	(4.41, 106.54)
Vaginal tears								
no	-		1.00		1.00		1.00	
yes	-		3.37	(0.59, 19.41)	1.70	(0.79, 3.67)	2.27	(0.94, 5.50)
Fetal weights								
< 3500 mg	-		1.00		1.00		1.00	
Macrosomia (> 3500 mg)	-		1.35	(0.85, 2.15)	1.59	(1.01, 2.50)	0.99	(0.53, 1.88)
Length of 1st and 2nd stage								
< 1 hour	-		1.00		1.00		1.00	
1 - 2.9 hours	-		2.42	(1.15, 5.06)	2.45	(1.14, 5.26)	1.95	(0.62, 6.15)
3 - 4.9 hours	-		3.25	(1.62, 6.53)	3.77	(1.86, 7.65)	4.93	(1.55, 15.70)
5 + hours	-		2.66	(1.22, 5.82)	3.12	(1.44, 6.74)	5.75	(1.73, 19.14)
AMTSL								
Yes	-		1.00		-		-	
No	-		9.44	(5.57, 16.01)	-		-	
AMTSL components								
Use of uterotonics in 3rd stage								
yes	-		-		1.00		1.00	
no	-		-		9.75	(4.58, 20.77)	11.26	(3.91, 32.47)
Early cord clamping								
yes	-		-		1.00		1.00	
no	-		-		1.36	(0.31, 6.04)	3.08	(0.52, 18.43)
Cord traction								
yes	-		-		1.00		1.00	
no	-		-		1.22	(0.21, 7.06)	8.26	(1.02, 67.00)
Uterine massage								
yes	-		-		1.00		1.00	
no	-		-		4.47	(0.94, 21.33)	0.64	(0.07, 5.58)

In the full multivariable model (Model 4), which included both ante- and intra-partum variables, elevated number of ANC visits (2-3 visits OR = 5.89, 95% CI 1.13-30.62; 4+ visits OR = 7.43, 95% CI 1.44-38.46), history of PPH (OR = 305.14, 95% CI 48.02-1938.87), anemia (OR = 2.73, 95% CI 1.43-5.23), labor augmentation (OR = 2.37, 95% CI 1.14-4.93), retained placenta (OR = 21.68, 95% CI 4.41-106.54), and length of 1^st ^and 2^nd ^stage (3-4.9 hours: OR = 4.93, 95% CI 1.55-15.70; 5+ hours: OR = 5.75, 95% CI 1.73-19.14) remained significantly associated with increased risk of PPH. Non-use of uterotonics in the 3^rd ^third stage (OR = 11.26, 95% CI 3.91-32.47), and cord traction (OR = 8.26, 95% CI 1.02-67.00) were the only components of AMSTL that remained significant in the full model.

**Figure 1:**
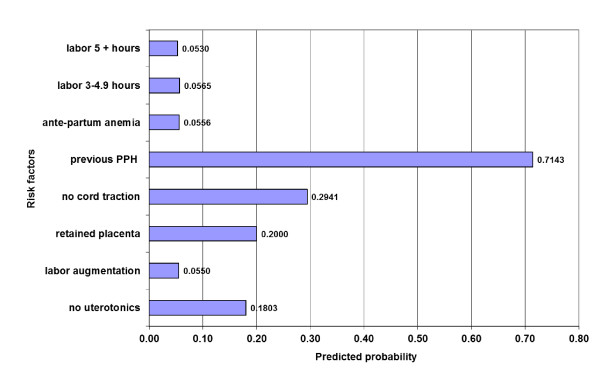
**Predicted probability for risk factors significantly associated with PPH**.

We used post model probability estimates to predict the probability of developing PPH with each one of the statistically significant risk factors in Model 4 (Figure 1). History of PPH, non-use of cord traction, and retained placenta each had a predictive probability of 20% or higher.

**Figure 2:**
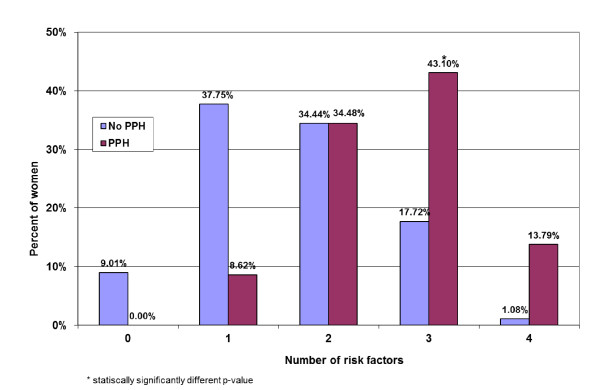
**Number of statistically significant risk factors for PPH among women with vaginal deliveries**.

**Figure 3:**
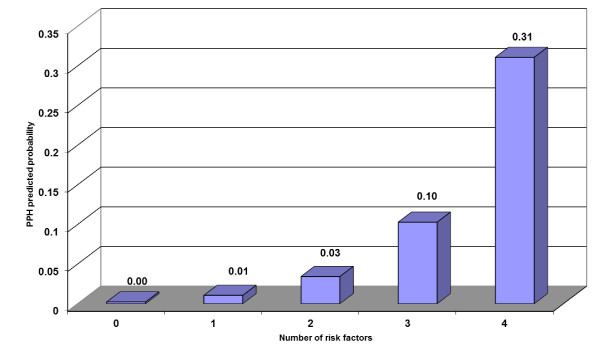
**Predicted probability of developing PPH according to number of statistically significant risk factors**.

We examined whether the percentage of women with 0, 1, 2, 3, or 4+ significant risk factors differed between those who had PPH and those who did not (Figure 2). Although percentages appear different for those with 0, 1, 3, and 4+ factors, the difference was only significant among women with 3 risk factors. Post model probability estimates showed that by screening and diagnosing women with three risk factors, we can identify 10% of women who will develop PPH (Figure 3). Similarly, screening for 4 risk factors may identify 31% of women who will develop PPH.

Table 3 presents the relative sensitivity, specificity, likelihood ratio, and percent correctly classified for women with 1, 2, 3, or 4+ risk factors for PPH. As the number of identified risk factors increases, so does percentage correctly classified. Perhaps because of the low incidence of PPH in the study population, the likelihood ratios are low for all risk factors. Women with 4+ identifiable risk factors had an LR of above 12.

**Table 3:** Specificity and sensitivity criteria for postpartum hemorrhage

Number of risk factors	Sensitivity (%)	Specificity (%)	Likelihood ratio	% correctly classified
1	100.00	9.01	1.10	12.66
2	91.38	46.76	1.72	48.55
3	56.90	81.20	3.03	80.22
4+	13.79	98.92	12.76	95.5

**Figure 4:**
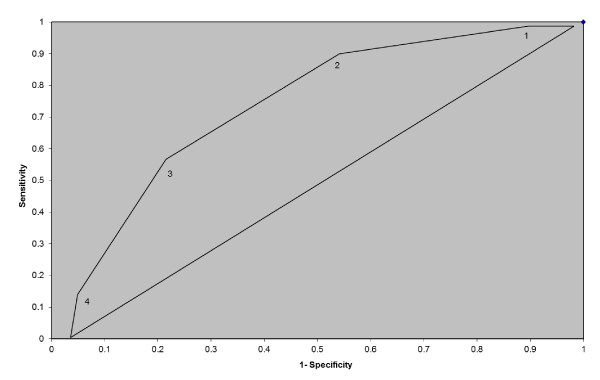
**ROC for number of risk factors**.

Figure 4 displays the relative utility of screening women with 1, 2, 3, or 4+ significant risk factors for PPH. Sensitivity, or the proportion of true positives, is high for using a screening cut point of 1 or 2 risk factors. However, the specificity, or proportion of true negatives, is low at 9% and 47% respectively. This means that if 1 risk factor was used as a cut point, 100% of those who would go on to have PPH would be captured. However, only 9% of those identified as positive would actually be positive. Thus you would have many people who would be screened as at risk who in reality may not be. Increasing the cut point to 2 risk factors would decrease sensitivity only marginally, but would increase specificity to 47%. Similarly, using 3 risk factors as a cut point would result in few false positives, but would also decrease sensitivity, meaning that some cases would be missed.

## Discussion

Results of this study show that most demographic and ante-partum risk factors have little association with development of PPH, with the exception of history of PPH, low hemoglobin, and high usage of ANC. In addition, the predictive probability of ante-partum and intra-partum risk factors for PPH is low, although women with four or more identified risk factors had a predictive probability of over 30%. This probability is higher than those observed by Tsu in Zimbabwe, and indicates that perhaps prediction of PPH is more significantly influenced by ante-partum variables [28].

The results of this analysis confirm our hypothesis that in vaginal, singleton deliveries, both ante-partum and intra-partum factors are associated with the incidence of PPH, but these associations are not predictive. Predictive probability of PPH based on any risk factor remains low. The exception is history of PPH, which had a predictive probability of 70% for incidence of PPH. A high number of antenatal care visits was also associated with increased risk, likely because this serves as a marker for high-risk pregnancies. The utility of screening based on number of risk factors should be considered in conjunction with benefits of positively identifying all possible cases, and costs of over treating.

The findings of this study indicate that AMTSL significantly reduces the risk of PPH. AMTSL has been recommended as a routine management for vaginal deliveries in hospital settings [30,31], but it is rarely used in Egyptian hospitals. An analysis of the management of the third stage of labor in an Egyptian teaching hospital showed that only 15% of the women during the study period received correct AMTSL [32].

To our knowledge, this is the first analysis to examine the utility of each component of AMSTL separately. Our analysis of the components of AMTSL indicates that non-use of uterotonics in AMTSL had the strongest association with increased odds of PPH after adjustment. Cord traction was also significantly associated with PPH after controlling for other components. Uterine massage and early cord clamping, however, appear to have no independent effect on risk of PPH, which could be due to a lack of variability between each component. We investigated the variability of early cord clamping and cord traction and found that there were some women who received one and not the other. As a result, we decided to include both components in the model.

This touches on an overall limitation of this analysis. As many of the identified risk factors are correlated with one another, collinearity of the variables may have biased our inferences. This limitation would likely lead to conservative effect estimates, as we may be over controlling for related factors. In order to account for covariance, we used robust estimator of standard errors.

Low predictive probability values may have been the result of very low incidence of PPH in the study; the incidence of PPH in the study hospitals was 3.7%. Administration of uterotonics and AMTSL in the study hospitals likely contributed to the low rate of PPH.

Despite a 52% reduction in the Egyptian maternal mortality ratio (MMR) during the 1990s, PPH remained the main cause of death. According to the National Maternal Mortality Study [33], primary PPH alone was responsible for 27% of all maternal deaths, making it the single most important cause of maternal death in Egypt. High parity, low socio-economic status of women, lack of skilled birth attendants, and mismanagement of PPH contribute to high maternal deaths in Egypt [34].

This study advances knowledge on the utility of screening for PPH in a clinical setting. This research has primary implications for screening and subsequent management of those at highest risk. Many of the identified risk factors cannot be prevented or altered, but women who present for delivery with these factors can be closely monitored, and when possible, intra-partum interventions that are significant risk factors should be avoided.

## Conclusion

While no single risk factor or combination thereof can reliably predict risk of hemorrhage after delivery, combinations of risk factors can be used for screening and management. Given the limited utility of screening for PPH, program planners and policy makers need to focus on the development and enforcement of protocols for prevention and treatment of PPH by considering the capacity and limitations of each context. The need for further research and policy work surrounding prevention efforts is especially important due to the low predictability of PPH.

## Competing interests

The authors declare that they have no competing interests.

## Authors' contributions

NP designed the initial intervention, conceptualized the analysis, conducted data analysis, and was the primary author for the original study and the current study. SH was the co-investigator for this study and the original study, and contributed to the conceptualization of the study. SB helped in the writing of several iterations of the manuscript, and prepared the manuscript for publication. DK participated in data analysis and interpretation of results. FV conducted the literature review regarding PPH risk factors. MH reviewed the data analysis and interpretation and revised the manuscript. All authors have read and approved the final manuscript.

## Pre-publication history

The pre-publication history for this paper can be accessed here:

http://www.biomedcentral.com/1471-2393/11/97/prepub
